# Successful Conversion Surgery after S-1 plus oxaliplatin Plus Zolbetuximab for Initially Unresectable Gastric Cancer with Pancreatic Invasion: A Case Report

**DOI:** 10.70352/scrj.cr.26-0392

**Published:** 2026-09-19

**Authors:** Yuma Fuse, Hidetaka Andrew Ono, Masanori Eguchi, Akiko Tamura, Seichiro Shirakabe, Ryo Fujiwara, Yusuke Yoshikawa, Naohiko Sugimura, Yukari Takata, Naoki Maeda, Mayu Kitamoto, Shohei Maruta, Susumu Daibo, Yohei Ota, Hirokazu Suwa, Kenichi Yoshida, Mayumi Yakeishi, Kazunori Nojiri, Takafumi Kumamoto

**Affiliations:** 1 Department of Surgery, Yokosuka Kyosai Hospital, Yokosuka, Kanagawa, Japan; 2 Department of Pathology, Yokosuka Kyosai Hospital, Yokosuka, Kanagawa, Japan

**Keywords:** gastric cancer, zolbetuximab, claudin 18.2, conversion surgery, S-1 plus oxaliplatin (SOX)

## Abstract

**INTRODUCTION:**

Zolbetuximab, a monoclonal antibody targeting claudin 18.2 (CLDN18.2), has recently demonstrated significant survival benefits when combined with chemotherapy for advanced gastric cancer. However, evidence regarding conversion surgery following SOX plus zolbetuximab therapy remains extremely limited.

**CASE PRESENTATION:**

A 59-year-old woman presented with epigastric discomfort. Endoscopy revealed a depressed lesion in the lower gastric body, and biopsy confirmed signet-ring cell carcinoma. Imaging and diagnostic laparoscopy indicated Type 4 gastric cancer with pancreatic invasion (cT4b, cN0, cP0, cCY0, cM0, cStage IVA). The tumor was HER2-negative, mismatch-repair proficient, PD-L1 combined positive score <1, and strongly positive for CLDN18.2. The patient received 13 courses of SOX plus zolbetuximab, resulting in radiological downstaging (ycT4a, ycN0, ycM0, ycStage IIB). It was determined that an R0 resection was feasible, and total gastrectomy with D2 lymphadenectomy and Roux-en-Y reconstruction was performed. Pathology revealed a minimal residual tumor (5 × 2 mm) with ypT2, ypN0, ypStage IB and a histological response of Grade 2b. R0 resection was achieved. The patient remains recurrence-free 7 months postoperatively.

**CONCLUSIONS:**

To our knowledge, this is the first published case describing successful conversion surgery following SOX plus zolbetuximab therapy. This case suggests that CLDN18.2-targeted therapy may facilitate curative resection in selected patients with initially unresectable gastric cancer.

## Abbreviations

CLDN18.2
: claudin 18.2
HER2
: human epidermal growth factor receptor 2
PD-L1
: programmed death-ligand 1
SOX
: S-1 plus oxaliplatin

## INTRODUCTION

CLDN18.2 is a tight junction protein that is selectively expressed in gastric epithelial cells and frequently retained during malignant transformation. Zolbetuximab, a monoclonal antibody targeting CLDN18.2, induces antitumor effects through antibody-dependent cellular cytotoxicity and complement-dependent cytotoxicity.^1,2)^

Recent phase III trials, including SPOTLIGHT and GLOW, demonstrated significant improvements in progression-free and overall survival when zolbetuximab was combined with fluoropyrimidine-platinum chemotherapy in patients with CLDN18.2-positive, HER2-negative advanced gastric cancer.^1,2)^

The C-SOLVE trial is currently evaluating zolbetuximab plus SOX as first-line therapy^3)^; however, clinical data regarding surgical outcomes or conversion surgery after this regimen are lacking.

We report a rare case of initially unresectable gastric cancer with pancreatic invasion that achieved successful R0 resection following SOX plus zolbetuximab therapy.

## CASE PRESENTATION

A 59-year-old woman presented with epigastric discomfort. Upper gastrointestinal endoscopy revealed a depressed lesion on the lesser curvature of the lower gastric body. Biopsy confirmed signet-ring cell carcinoma.

**Fig. 1:**
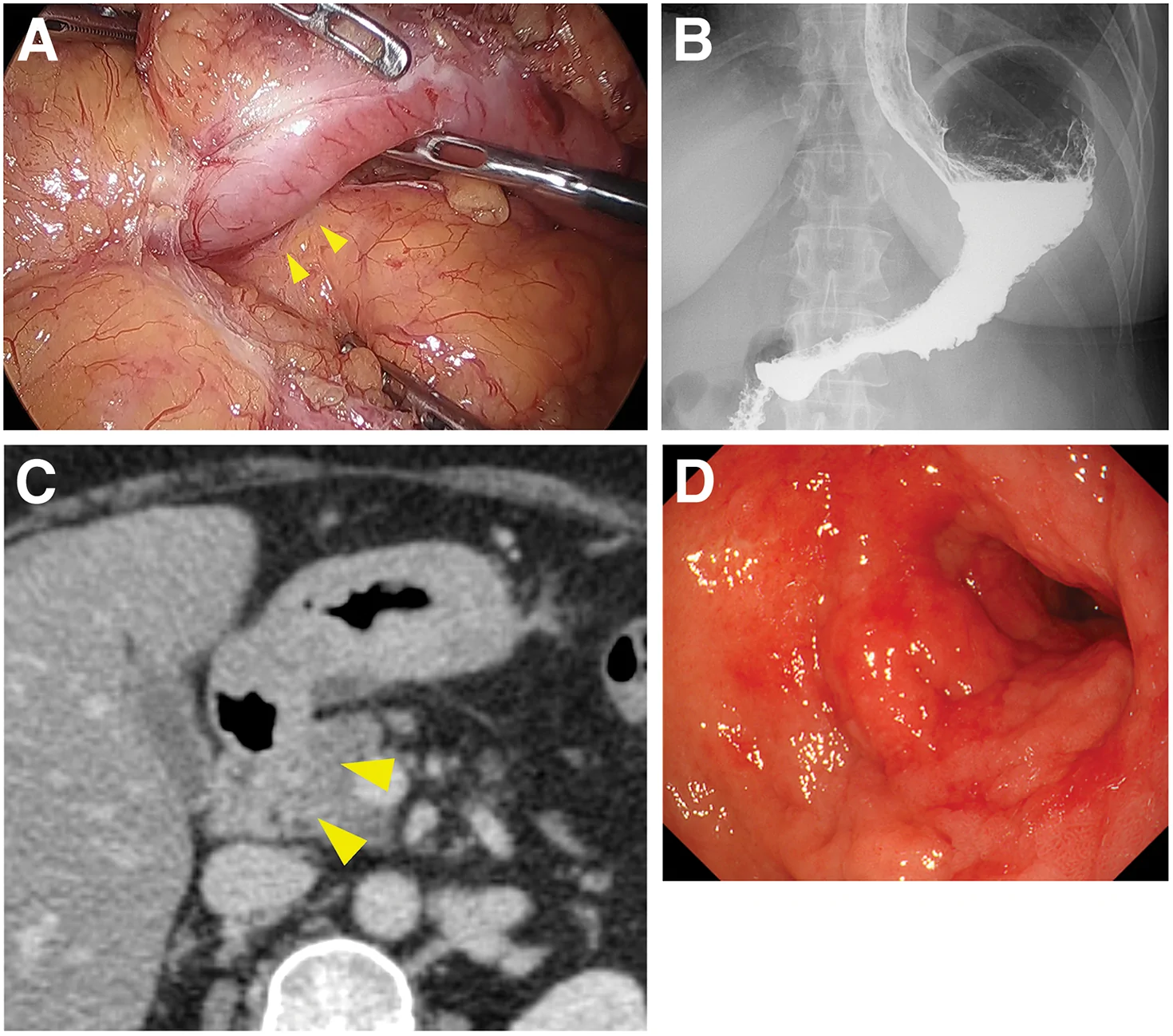
Pre-treatment findings. (**A**) Diagnostic laparoscopy demonstrated direct pancreatic invasion (arrow). (**B**) Upper gastrointestinal series demonstrated diffuse wall thickening and marked rigidity of the lower gastric body with poor distensibility. The gastric lumen appeared narrowed without a distinct mass lesion, findings consistent with type 4 gastric cancer. (**C**) Contrast-enhanced CT before chemotherapy demonstrating direct pancreatic invasion (arrowheads). (**D**) Upper gastrointestinal endoscopy before chemotherapy showing diffuse wall thickening and poor distensibility of the lower gastric body, consistent with type 4 gastric cancer.

Contrast-enhanced CT demonstrated direct invasion of the pancreas, and diagnostic laparoscopy confirmed pancreatic invasion without peritoneal dissemination or positive cytology (**Fig. 1**). The clinical diagnosis was type 4 gastric cancer, cT4b (serosal invasion: pancreas), cN0, cP0, cCY0, cM0, cStage IVA, according to Japanese Classification of Gastric Carcinoma (16th edition).^4)^ This tumor showed HER2 negativity, mismatch-repair proficiency, PD-L1 combined positive score (CPS) <1, and strong CLDN18.2 expression. CPS was evaluated using the PD-L1 IHC 22C3 pharmDx assay.

### Systemic therapy

The patient received 13 cycles of SOX plus zolbetuximab. (S-1: 120 mg/day on days 1–14 every 3 weeks, Oxaliplatin: 100 mg/m^2^ on day 1, Zolbetuximab: 800 mg/m^2^ in cycle 1, then 600 mg/m^2^ thereafter).

**Fig. 2:**
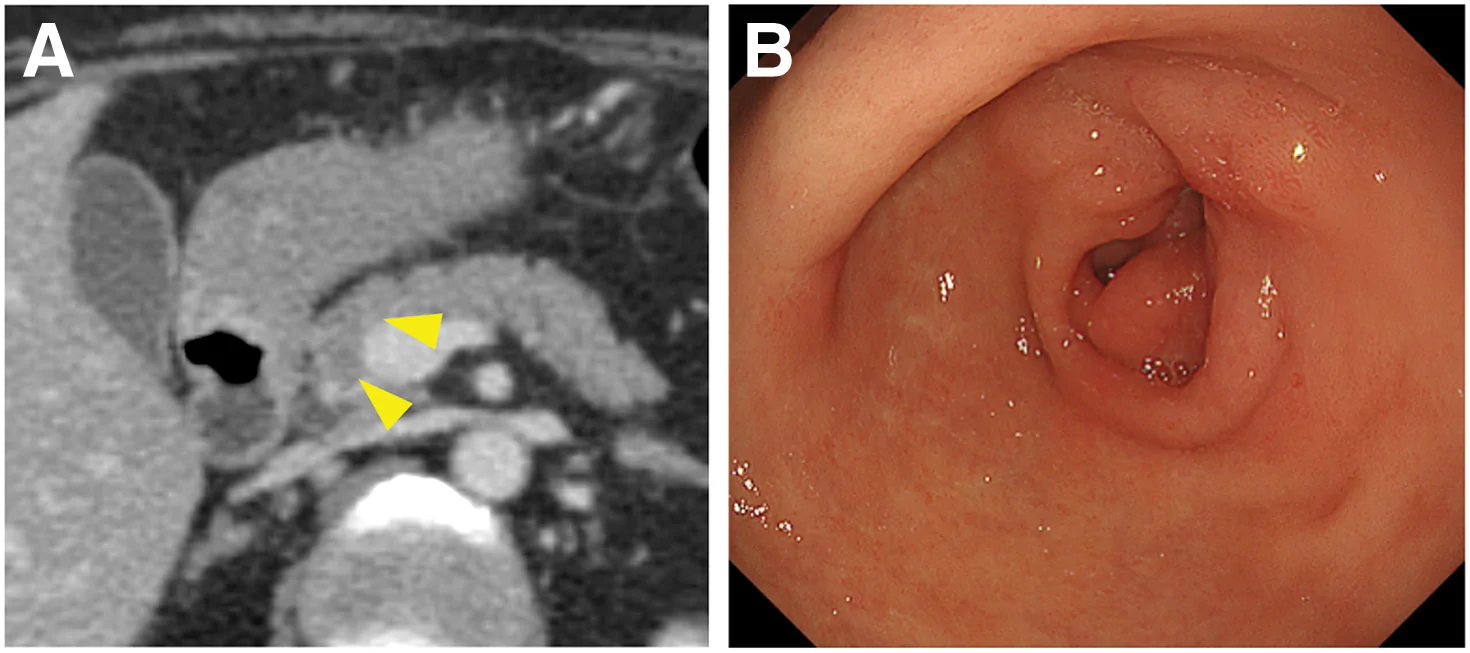
Response to chemotherapy. (**A**) Contrast-enhanced CT after chemotherapy demonstrating marked regression of the pancreatic invasion (arrowheads). (**B**) Upper gastrointestinal endoscopy after chemotherapy showing marked regression of the type 4 lesion, with improved luminal distensibility and residual ulcerative changes.

Treatment was well tolerated. Radiological evaluation showed tumor shrinkage and downstaging to ycT4a, ycN0, ycM0, ycStage IIB (**Fig. 2**).

Before chemotherapy, the serum tumor marker levels were carcinoembryonic antigen (CEA) 0.4 ng/mL, carbohydrate antigen 19-9 (CA19-9) 8.8 U/mL, and CA125 43.6 U/mL. Following chemotherapy, the corresponding levels were 1.8 ng/mL, 13.7 U/mL, and 12.3 U/mL, respectively. Although CEA and CA19-9 remained within the normal range, the elevated CA125 level normalized after chemotherapy.

### Surgery

Conversion surgery was performed. Although laparoscopic distal gastrectomy was initially considered, total gastrectomy was required due to suspected proximal margin involvement. D2 lymphadenectomy and Roux-en-Y reconstruction were performed.

### Pathological findings

Pathology revealed a minimal residual tumor measuring 5 × 2 mm. Final staging was ypT2, ypN0 (0/26), ypStage IB, with a histological response of Grade 2b.

**Fig. 3:**
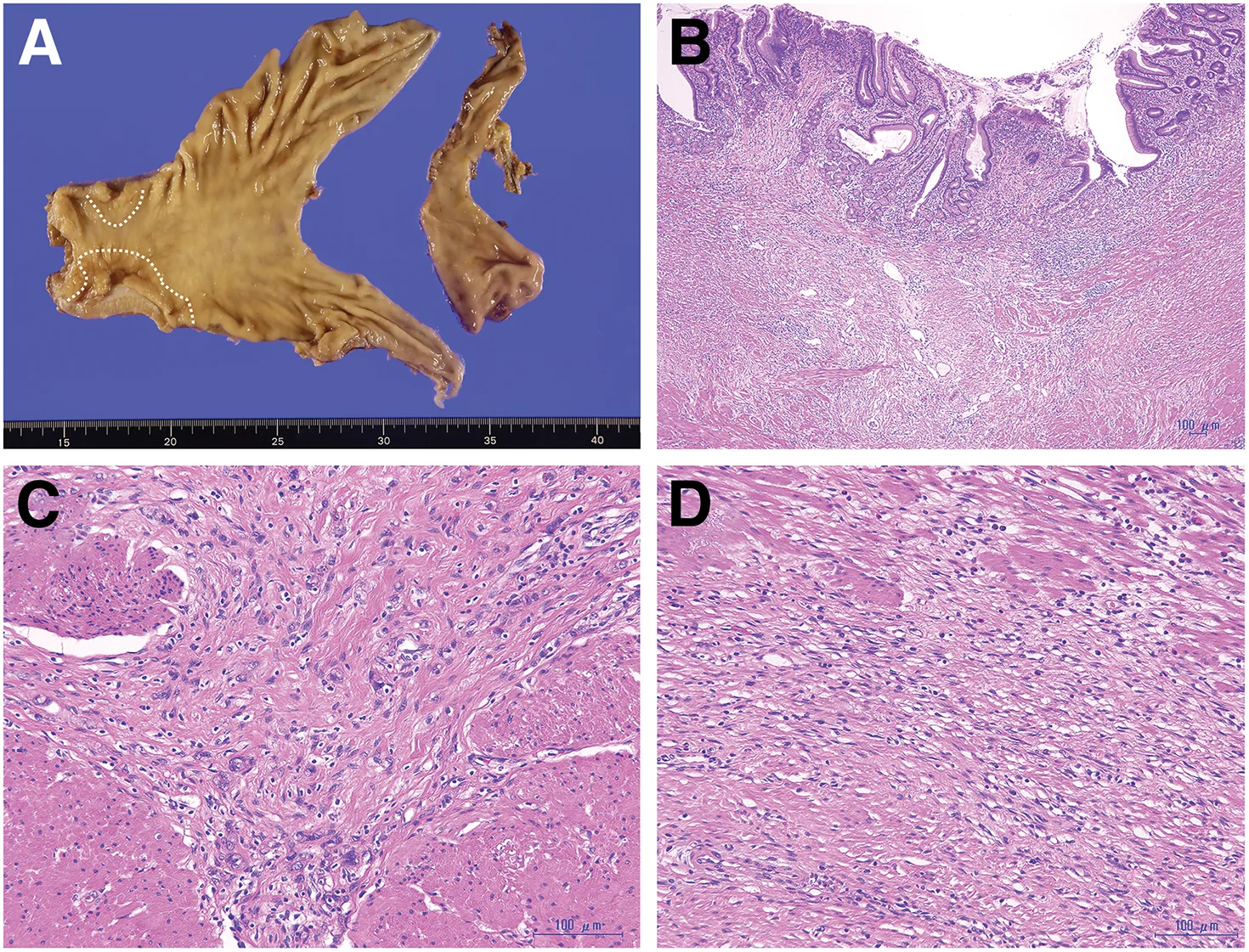
Pathological findings of the resected specimen. (**A**) Macroscopic findings of the resected stomach. (**B**) Low-power histopathological findings. (**C**) Residual tumor cells were confined to the ulcer base. (**D**) No viable tumor cells were identified at the raised ulcer margin.

An R0 resection was achieved (**Fig. 3**).

The postoperative course was uneventful. Adjuvant chemotherapy was not administered because pathological examination demonstrated only minimal residual disease, and after discussion with the patient, careful observation was selected. The patient remains recurrence-free 7 months after surgery.

## DISCUSSION

Zolbetuximab has recently emerged as a key therapeutic option for CLDN18.2-positive gastric cancer, with 2 phase III trials demonstrating clear survival benefits.^1,2)^ Despite these advances, evidence regarding conversion surgery after zolbetuximab-based chemotherapy remains extremely limited. The marked antitumor activity observed in these trials suggests that zolbetuximab-based chemotherapy may increase the opportunity for conversion surgery in selected patients with initially unresectable disease.

To our knowledge, no prior reports have specifically described conversion surgery following SOX plus zolbetuximab, although the ongoing C-SOLVE trial may provide future insights.

At the time treatment began, there was still no evidence from prospective studies supporting the combination of SOX and zolbetuximab. However, SOX is one of the standard first-line treatments for advanced gastric cancer in Japan, and at our hospital, it was routinely used as the institution’s standard chemotherapy regimen for HER2-negative advanced gastric cancer. Therefore, considering that the patient’s tumor was CLDN18.2-positive, we selected SOX as the backbone of chemotherapy. Following a multidisciplinary consultation and after obtaining the patient’s informed consent, we opted for the SOX plus zolbetuximab regimen.

The present case was classified as cStage IVA according to Japanese Gastric Cancer Association.^4)^ Curative resection at the time of diagnosis would have required pancreatoduodenectomy (PD) to achieve an R0 resection because of direct pancreatic invasion. A previous review suggested that PD for gastric cancer should be reserved for carefully selected patients because of its high invasiveness and the limited evidence supporting its oncologic benefit.^5)^ Therefore, initial systemic chemotherapy was selected, and surgery was considered only after a favorable treatment response. Accordingly, the present case was considered eligible for conversion surgery according to the definition proposed by Yoshida et al.^6)^

SOX plus zolbetuximab induced marked tumor regression, enabling curative resection despite the initial pancreatic invasion. The pathological findings of minimal residual disease and a Grade 2b response suggest substantial chemosensitivity.

This case highlights the potential role of CLDN18.2-targeted therapy in expanding surgical opportunities for patients with initially unresectable gastric cancer. Further prospective studies are warranted to define optimal patient selection and timing for conversion surgery; however, our experience suggests that zolbetuximab-based chemotherapy may facilitate curative-intent surgery in carefully selected patients with CLDN18.2-positive gastric cancer.

Furthermore, recent studies have emphasized the importance of conversion surgery in selected patients with initially unresectable gastric cancer.

Yoshida et al. proposed a biological classification system for conversion therapy in stage IV gastric cancer.^6)^

Yoshida et al. and Yasufuku et al. reported that conversion surgery after chemotherapy is safe and can achieve favorable long-term survival in selected patients with advanced gastric cancer, particularly when R0 resection is achieved, with outcomes strongly influenced by patient selection and prognostic factors.^7,8)^

## CONCLUSIONS

SOX plus zolbetuximab therapy may provide an opportunity for conversion surgery in selected patients with initially unresectable gastric cancer. This case represents the first documented example of successful R0 resection following this regimen.

## DECLARATIONS

### Availability of data and materials

The datasets supporting the conclusions of this article are included within the article.
